# Dental Convention

**Published:** 1862-05

**Authors:** 


### DENTAL CONVENTION.

On another page will be found the notice of the next meeting of the American Dental Convention, together with the order of business.

These Conventions are important and of much advantage to the profession. This is perhaps the best place to compare notes, and modes of practice: this, with the cultivation of acquaintance and professional sociality constitute, as we conceive, the chief function of this kind of meetings. But all these are very important, as any one will readily concede.

Whatever the attainments of any may be, it will certainly be advantageous to compare himself with others, by this means only can he know whether he is up to the full measure of professional attainments. The discussion of practice comes prominently within the sphere of this Convention: and it would perhaps be quite as well, were there less reaching out constantly after *new* things, or rather, more thorough consideration of things and modes that are already in use. Is it not true that many of these are susceptible of still farther development, and higher spheres of usefulness? Let us all then resolve to go up to these meetings prepared to add something to the common stock: to *give out* as well as *absorb.* T.

